# Spontaneous stereotypes: revisiting stereotype dimensions through language

**DOI:** 10.3389/fpsyg.2026.1727409

**Published:** 2026-04-02

**Authors:** Alessandra Caldera, Cristina O. Mosso

**Affiliations:** Department of Psychology, University of Turin, Turin, Italy

**Keywords:** natural language, social evaluation, social inequality, spontaneous stereotypes, status, stereotype content

## Abstract

**Introduction:**

The present study, grounded in the Spontaneous Stereotype Content Model, explores the extent to which the evaluation of one group reflects the vertical and horizontal facets of social evaluation and stereotypes.

**Methods:**

In an online survey, 245 Italian youths (60.4% female; *M*_age_ = 26, *SD* = 3.4) indicated the most common words used to describe Southern Italians in Italy, through free-text responses and scale-based measures.

**Results:**

Southern Italians are perceived as more sociable, but less capable, assertive, and moral. The four facets of social evaluation reflect the spontaneous stereotypes detected through natural language analysis.

**Discussion:**

The implications of positive stereotypes about social groups in language and communication are discussed. Ambivalent stereotypes can potentially minimize the perceived impact of negative traits, thereby perpetuating socio-economic inequalities.

## Introduction

Stereotypes emerge in natural language and are shared in a social world (Beukeboom and Burgers, 2019). Research on stereotypes grounded in the Stereotype Content Model (Fiske et al., 2002) consistently shows that their content refers to two fundamental dimensions, warmth and competence, and is predicted by group status, defined as economic success, educational level, and professional prestige, and intergroup relations. The higher the status of a social group, the higher the degree of competence attributed to its members, whereas the extent to which they are perceived as cooperative or competitive is associated with, high or low warmth, respectively (Fiske, 2018). Warmth and competence attributions result in various patterns of stereotypes (e.g., warm and competent, warm but incompetent) (Fiske et al., 1999). Higher-status group members are evaluated as more competent and ambitious, but less warm, generous and friendly. The opposite pattern is observed for lower-status members (Yzerbyt, 2016). Positive traits can be employed to compensate for negative traits in another dimension, to favor one’s own group, either to protect it or to assert its superiority, without appearing discriminatory (Cambon and Yzerbyt, 2018). In these terms, attributing competence, efficiency and ambition to high-status groups can reinforce the belief that social positions are deserved (Oldmeadow and Fiske, 2007; Jost and Banaji, 1994).

This study aims to examine the stereotypes of a real-world social group. Through a cross-sectional design, we investigated how stereotypes are shared through language within a sociocultural context. Research on stereotypes and status has been mostly anchored to the two-dimensional approach, while a multifaceted approach is recommended (Yzerbyt et al., 2025). A comprehensive review of social evaluation models (Abele et al., 2021) suggests that the relevance of sub-dimensions depends on the type and number of evaluation targets, and the evaluation’s purpose. Although the two broader dimensions can be sufficient to evaluate multiple targets, social judgments of a single target are more detailed and accurate. Recent findings suggest that spontaneous stereotypes are distributed over more than two clusters of traits (Nicolas et al., 2022).

Therefore, the Vertical and Horizontal Model of Social Evaluation and the Spontaneous Stereotype Content Model (SSCM; Nicolas et al., 2022) were applied to the stereotypes of Southern Italians, investigating their content through the analysis of both free-text responses and scale-based measures of the four facets of social evaluation.

The vertical dimension of social evaluation refers to the target’s social standing and consists of two facets: ability and assertiveness. Ability is inherently defined by perceived competence and effectiveness, whereas assertiveness denotes the perceived ambition to achieving goals and self-confidence. The horizontal dimension represents benevolence and trust. It encompasses morality, the intentional facet related to honesty and reliability, and sociability, the emotional facet related to warmth and friendliness (Abele et al., 2016).

A comprehensive analysis of stereotypes concerning Southern Italians across the four facets of social evaluation and their association with socio-structural variables can offer novel insights into the relationships between stereotypes and inequality for several reasons.

Firstly, social inequality in Italy is evidenced by The North–South divide, known as the “Southern Question” (Gramsci, 1995). Disparities in socioeconomic status, resources distributions, and educational and career opportunities between northern and southern regions led to migratory flows since 1861. Over the past two decades, approximately 2.5 million individuals have moved from southern Italy, 81% of whom have migrated to northern Italy. The most recent migratory flow primarily concerns young adults (Svimez, 2023).

Secondly, according to previous studies, stereotypes of Northern and Southern Italians are consistent with the SCM hypotheses. Northern Italians are typically perceived as more competent and higher- status, while Southern Italians are viewed as warmer and lower-status (Durante et al., 2009; Jost et al., 2005). Moreover, these studies have shown that the content of stereotypes and perceived status of Southern Italians are shared between the ingroup and the outgroup (see also Villano and Passini, 2018). To date, sub-dimensions of stereotypes of this group have not been investigated.

Thirdly, examining the facets of the vertical and horizontal dimensions of stereotypes of Southern Italians would offer new insights into the role of ability and assertiveness in socioeconomic status. Assertiveness appears to be more closely associated with status than ability (Yzerbyt et al., 2022), reflecting the individualistic values of Western societies. Individuals socially learn to associate higher status with those who demonstrate commitment to goal achievement and exhibit traits consistent with these values (Cambon, 2022). Despite this evidence, relatively few studies in this area of research have directly investigated the relationship between the status of real groups and facets of social evaluation.

Stereotypes associated with Southern Italians are investigated through the analysis of free-text responses and scale-based measures of social attributions, in a sample of Italian young adults. Prior studies on stereotypes of Southern Italians employed scale-based measures (Durante et al., 2009; Jost et al., 2005). Thus, the SSCM is applied to explore how stereotypes are shared in Italian society through language, emphasizing the relevance of the context of evaluation (e.g., nations) in shaping their content. The analysis of free-text responses is conducted across the facets of social evaluation, as well as in terms of their directionality (high/low) and valence (positive/negative), using a semi-automated dictionary, which showed to cover 80% of words used to describe major social groups (Nicolas et al., 2021).

Although the purpose of this study is exploratory, based on prior research we hypothesize that:

*H1*: Southern Italians would be evaluated (H1a)—and would evaluate themselves (H2b)—as high on the horizontal facets of social evaluation (sociability and morality), and low on the vertical facets (ability and assertiveness), as well as on status.

*H2*: No significant differences between groups would emerge in stereotypical attributions.

*H3*: The content of stereotypes would exhibit a four-factors structure.

## Methods

Two hundred and forty-five Italian young adults (60.4% female, 39.6% male; age range: 18–35 years; *M_age_* = 26, *SD* = 3.4) from Southern (41.6%), Northern (23.5%) and Central Italy (35%) were recruited through the snowball technique to participate in the study via a Qualtrics online survey. Due to historical, geographical and economic factors, Italy is conventionally divided into three macro-areas, reflecting the structural differences before and after the unification of the nation. Consequently, the categorization of groups for the analysis of stereotypes was informed by this classification, to observe potential differences or their absence, despite the socio-economic disparity between the northern and southern regions.

A minimum sample size of 159 participants for a one-way ANOVA for differences between 3 groups with a medium effect (0.25) *α* = 0.05 and power 0.80 was obtained as required using G* power (Faul et al., 2009).

Participants were asked to name common words used to describe people from southern Italy. They were informed that we were not interested in their personal opinions, but rather in the beliefs that were shared within their society (Fiske et al., 2002). Thus, they indicated how most people in their society view Southern Italians compared to Northern Italians, using 12 traits to measure the four facets of stereotypes (Nicolas et al., 2021) on a 5-point bipolar scale. Three items per facet were employed to measure stereotypes on sociability (e.g., cold/warm; hostile/friendly), morality (e.g., dishonest/honest; unreliable/reliable), ability (e.g., incompetent/competent; uneducated/educated) and assertiveness (e.g., doubtful/determined; dependent/independent).

Following previous studies (Durante et al., 2009), perceived intergroup relations (3 items; e.g., “A cooperative relationship exists between southerners and northerners”) and perceived status inferiority in terms of economic success (ES), professional prestige (PS), and educational level (LS) compared to Northern Italians (3 items; e.g., “Generally, southerners tend to obtain less prestigious job positions than northerners”) were included to examine their relationship with the vertical and horizontal facets.

Participants provide information about age, nationality, educational degree, and occupational status and geographical area. Finally, seven items from the Brief Sense of Community Index (Peterson et al., 2008) to control social identification.

## Results

*Content warning: please note that this section contains content of stereotypes that may be found upsetting.*

The content of the spontaneous stereotypes was coded using the SSCM dictionary via the *R package SADCAT* (Nicolas et al., 2021; see Supplementary materials). The semi-automatized text analysis adhered to the steps outlined in the procedure.^1^

Only participants who provided at least two words were selected for analysis (*N* = 204; 60.8% female, 39.2% male; *M_age_* = 26.3 years, *SD* = 3.33). The Italian free responses were translated into English and then back-translated. A binary code (0 = no; 1 = yes) was assigned to each word based on its presence in the dictionary for each variable (e.g., “sociability,” “sociability high,” “sociability low,” “sociability positive,” “sociability negative”).

Eight hundred and twenty-one words were counted, 40.7% of which were used by Southern Italians, 25.4% by those from the north and 33.9% by those from central Italy. Unmatched words were coded according to their etymological meaning (see Supplementary materials). The coding was performed independently, and the agreement was assessed (*r* = 0.90). For example, the word “*mafia*” was coded into the category of morality, specifically as low directionality, or the word “*terrone*” as low ability as related to ignorance and limited competence (Viola, 2019).

48.2% of the words were coded as negative, 31.3% as positive and 20.5% as neutral for their general valence. The most frequent negative adjectives were “*terrone*” (n = 92), “*ignorant*” (n = 40) “*mafia*” (n = 32) and “*idle*” (n = 30), while the positive adjectives were “*warm*” (n = 47), “*welcoming*” (n = 40), “*cheerful*” (n = 27) and “*generous*” (n = 17). The most frequent words are reported in upplementary materials.

90% of responses were related to the four facets of social evaluation and showed that 248 words were coded in the clusters Sociability (30.2%), 208 in Morality (25.3%), 186 in Ability (22.6%) and 107 in Assertiveness (13%). The additional clusters of social evaluation related to Status and Beliefs (Koch et al., 2016) appeared with 23 (2.9%) and 43 (5.2%) words, respectively. Some words were coded in the other clusters of the dictionary (e.g., Emotion, Geography, Occupation). The main analyses focused on the four facets of social evaluation, additionally including Status in descriptive statistics. Some of the words were coded in more than one cluster due to dimensional overlap (see upplementary materials).

**Figure 1:**
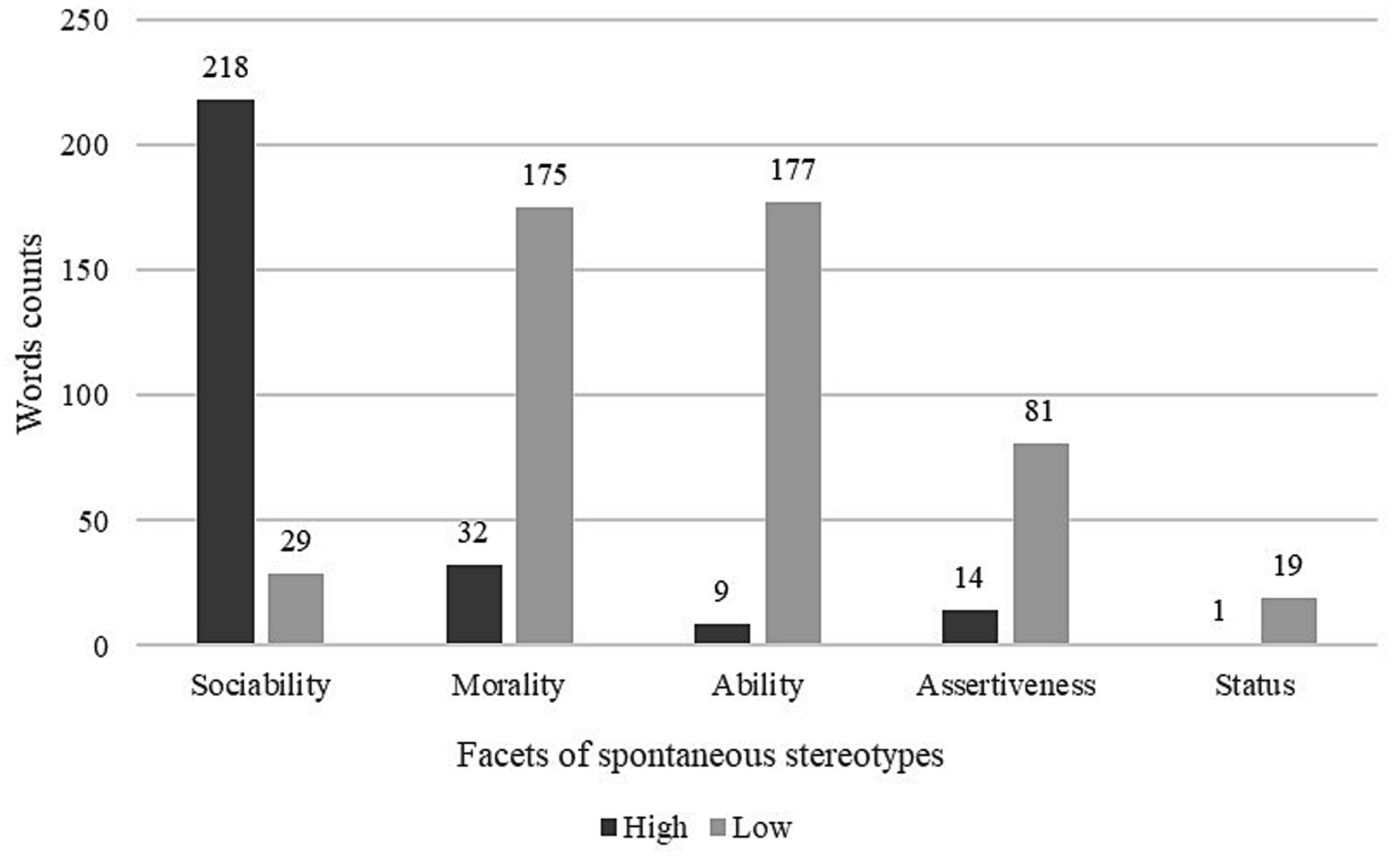
Word counts, separated by each facet of stereotypes and directionality (high/low). Some words coded within the broad category are not coded as denoting high/low directionality.

Descriptive analyses were conducted (H1), showing that Southern Italians were spontaneously stereotyped as high in Sociability and low in Ability, Assertiveness, Morality and Status (Figure 1). The words related to high sociability were the most frequently used by participants.

To examine differences between geographical groups (H2), separate binomial generalized linear models were tested for each of the four facet (e.g., “sociability”) and its directionality (e.g., “sociability high,” “sociability low”). A weak difference emerged in the likelihood of using words related to low morality (LRT *χ*^2^ = 17.5 (2), *p* < 0.001; *R*^2^ = 0.02) and to low sociability (LRT *χ*^2^ = 13.5 (2), *p* = 0.001, *R*^2^ = 0.05). Southern Italians were less likely to use words related to low morality than participants from North (*z* = −3.56, *p* = 0.001) and Central Italy (*z* = −3.53, *p* = 0.001). Unexpectedly, participants from Central Italy were less likely to use words referring to low sociability than Northern Italians (*z* = 2.89, *p* = 0.01). However, no significant differences between groups were observed in the frequency of words denoting high sociability (LRT *χ*^2^ = 0.74 (2), *p* = 0.691).

Spearman’s correlations between words counts per category showed a significant positive association between the use of words related to morality and assertiveness (*ρs* = 0.241, *p* < 0.001) and those related to morality and ability (*ρs* = 0.154, *p* = 0.03), consistent with the content of stereotypes. Additionally, the word count of ability and sociability emerged as negatively correlated (*ρs* = −0.321, *p* < 0.001), in line with the empirical findings on the compensation effect based on group status (Cambon and Yzerbyt, 2018). Unexpectedly, ability and assertiveness correlated negatively (*ρs* = −0.379, *p* < 0.001). Participants may have spontaneously emphasized one dimension while downplaying the other (Yzerbyt et al., 2022).

The analysis of words used first by each participant, the “Ability low” cluster was the most salient (n = 86), followed by “Sociability high” (n = 54).

The analysis of the scale-based stereotypes was performed with Jamovi 2.3.28 (The Jamovi Project, 2024). All participants were included. To address H3, comparing the multifaceted theoretical model of stereotypes to the two-dimensional one, a confirmatory factor analysis (CFA) for the four-factors structure (sociability, morality, ability and assertiveness) was conducted with Mplus 8 (Muthén and Muthén, 2017) and showed a good fit of the model (*χ*^2^ (48) = 60.64, *p* = 0.104; CFI = 0.977; SRMR = 0.051; RMSEA = 0.033, RMSEA 90% CI [0.000, 0.056]). In contrast, the CFA for the vertical and horizontal two-factorial structure did not show a good fit (*χ*^2^(53) = 249.2, *p* < 0.001; CFI = 0.640; SRMR = 0.124; RMSEA = 0.123, RMSEA 90% CI [0.108, 0.138]), confirming H3. Two items (“*competitive*” and “*confident*”) were excluded as their factor loadings did not show significance. The final model showed an improved fit (*χ*^2^(29) = 26.95, *p* = 0.574; CFI = 1.00; SRMR = 0.038; RMSEA = 0.000, 90% CI [0.000, 0.044]).

Descriptive analysis testing H1 showed that Southern Italians were stereotyped as high in Sociability and low in Ability, Morality and Assertiveness, similarly to the results of the open answers (Table 1). Southern Italians were evaluated favorably exclusively on sociability, whereas morality attributions were found to be lower.

**Table 1:** Means, standard deviations, and correlations between scale-based measures (*N* = 245).

Variable	M (SD)	1	2	3	4	5	6	7
1. HS	4.45 (0.61)	–						
2. HM	2.72 (0.80)	0.182^**^	–					
3. VC	2.16 (0.82)	0.071	0.437^***^	–				
4. VA	2.87 (0.81)	0.060	0.201^**^	0.404^***^	–			
5. PS	3.35 (1.04)	0.071	0.016	−0.076	−0.140^*^	–		
6. ES	3.80 (0.87)	0.093	0.013	−0.118	−0.111	0.244^***^	–	
7. LS	3.26 (1.16)	−0.032	−0.071	−0.066	−0.044	0.202^*^	0.128^*^	–
8. CI	2.88 (0.61)	0.005	0.126^*^	0.234^***^	0.105	0.155^*^	0.117	−0.050

^*^p < 0.05; ^**^p < 0.01;^***^p < 0.001. HS, Horizontal-Sociability; HM, Horizontal-Morality; VC, Vertical-Ability; VA, Vertical-Assertiveness; PS, fewer professional prestige; ES, fewer economic success; LS, fewer educational level; CI, cooperative intergroup relations.

Pearson’s *R* correlations showed that relationships between facets reflects the model’s structure and the content of Southern Italians stereotypes.

A negative relationship between assertiveness and status inferiority related to professional prestige by Southern Italians emerged. However, this relationship appears marginal, and no associations have been found with the other two measures of status.

Furthermore, participants who socially attributed more ability to Southern Italians, believe that the relations between Northern and Southern Italians is more cooperative. Nevertheless, no significant relationship emerged between perceived cooperative relations and sociability, whereas the correlation with morality was only marginally significant.

Separate general linear models were performed to examine the effect of group membership on each facet of stereotypes (H2). A weak significant effect of group membership on social attributions of morality was observed (*F*(2,240) = 6.39, *p* = 0.002, *partial η^2^* = 0.05). Southern Italians attributed more morality to their ingroup than Northern Italians (Bonferroni correction: *t* = 3.55, *p* = 0.001), in line with the results of the analysis of the spontaneous stereotypes.

## Discussion

The results showed that Southern Italians are socially perceived as warm but incompetent, non-assertive and immoral. The words used spontaneously matched the dictionary in terms of high sociability and low morality, ability and assertiveness. This proves stereotypes in natural language reflect the facets of social evaluation (Nicolas et al., 2022).

The present findings show partial consistency with those of earlier studies (Durante et al., 2009; Jost et al., 2005), adding novel insights. The observed discrepancy between sociability and morality underscores that stereotypes can vary between facets (Yzerbyt et al., 2025). Positive stereotypes of Southern Italians are predominantly exhibited in a single facet of social evaluation, specifically their sociability. Negative stereotypes on ability, assertiveness and morality have been found. These outcomes would have been obscured by a two-dimensional approach. The free-text analysis revealed a general tendency to spontaneously name positive stereotypes about sociability. Despite this, this category emerged second in order of appearance, following traits denoting low ability.

Furthermore, the relationship between status and assertiveness was consistent with the empirical findings on the relationship between status and assertiveness (Yzerbyt et al., 2022). However, it was only marginally significant and limited to professional status. Moreover, there was no evidence to suggest that status was associated with ability. This highlights the need for more in-depth investigation into the relationship between vertical stereotypes and status. Moreover, perceived cooperative intergroup relations were not related to sociability, whereas a relationship with ability emerged. These results are inconsistent with the SCM premises, which assume that intergroup relations predict horizontal stereotypes. Status is presumed to be linked to ability and assertiveness, and independent from cooperation/competition. However, the degree of intergroup cooperation can be positively influenced by perceived similarity of competence, and interdependence can increase the relevance of others’ vertical traits (Abele et al., 2021).

The findings indicate that stereotypes attributed to Southern Italians are broadly shared among both ingroup and outgroup members, reflecting the cultural nature of stereotypes. Southern Italians attributed more morality to their group than others and were less likely to use words denoting low morality, consistently with the empirical findings on the primacy of ingroup morality as a source of pride and self-esteem (Brambilla and Leach, 2014). However, the effect of group membership was only marginal.

The present study offers novel insights into the integrated social evaluation model in the analysis of societal stereotypes, especially those that arise spontaneously in language. The results highlight the potential implications of referring to positive stereotypes in conversations, especially in educational contexts, given that positive stereotypes have been demonstrated to subsequently activate the negative ones and inducing stereotype threat (Kahalon et al., 2018). Moreover, while protecting identity and avoiding discrimination, sociability traits are independent from status (Bareket and Fiske, 2023). Conversely, a stereotypical lack of ability and assertiveness has the potential to hinder social mobility within the social hierarchy for low-status groups, such as Southern Italians.

However, this study is subject to several limitations. The sample is exclusively composed of young Italian adults, recruited through snowball sampling, which limits the generalizability of the findings. The SCM originally adopted a multi-group perspective of analysis, whereas the integrated social evaluation model emphasizes the relevance of subdimensions when investigating single targets. Consequently, the focus was exclusively on Southern Italians as case study, while precluding intergroup comparisons and restricting the generalizability of the findings. Moreover, the spontaneous stereotypes were analyzed through a dictionary created and validated exclusively in English. Translation could have introduced semantic distortion. Consequently, an Italian validation of the SSCM dictionary is recommended for future research.

The consequences of stereotypes for young people should be addressed in future research to investigate the implications for social identity threat associated with vertical and horizontal stereotypes.

The analysis of stereotypes in natural language enables researchers to address stereotypes in a real social context as they are shared by individuals within society and strengthens the predictability of social evaluation models.

## Data Availability

The datasets presented in this study can be found in online repositories. The names of the repository/repositories and accession number(s) can be found at: Open Science Framework (https://osf.io/z967h/?view_only=30f146ea9b1c451a9f5cce89fe0db6de).

## Footnotes

1
https://github.com/gandalfnicolas/SADCAT
