# Supplementary material for: Spontaneous stereotypes: revisiting stereotype dimensions through language
Source: Front Psychol. 2026 Apr 2;17:1727409. doi: 10.3389/fpsyg.2026.1727409 (PMC13083014; doi:10.3389/fpsyg.2026.1727409)
Supplement: Supplementary file 1 [file Supplementary_file_1.docx]

Supplementary Material

# Supplementary Information on the Procedure

Qualitative coding was performed by two independent judges, namely one of the authors and a bilingual experimenter who was unfamiliar with the study rationales.

The automated coding of the words was conducted using the script available at the following link (<https://github.com/gandalfnicolas/SADCAT>), inside the "vignettes" folder (see "Text_Coding_Example_Guide.Rmd"). The dictionary employed for coding the words in the dataset is the complete "All.steps_Dictionaries.Rd" dictionary, which can be retrieved from the "man" folder. The procedural steps are outlined as follows: Install the required packages; Load the dictionary; Pre-process (e.g. transform to lower case); Lemmatize the words; Transform into a format suitable for coding as a dictionary; Create a dictionary from the original dataset; Load the dataset; Lemmatize the data; Match the words in the dataset to the dictionary; Calculate the valence (positive and negative words); Calculate the directionality (high and low); Calculate the no match; Create a new dataset containing the words numerically coded into variables (The _dic variables indicate the words coded into that dimension, regardless of directionality and valence. The _dic_hi and _dic_lo variables indicate the directionality of the words.The _dic_pos and _dic_neg variables indicate the valence of the words).

As words (not sentences or paragraphs) were entered into the loaded dataset, our variables are coded as binary (0 = no; 1 = yes).

Additional information can be accessed via the original source (see also Nicolas et al., 2021).

# Supplementary Information on the Analyses

Sensitivity analysis for binomial logistic regressions indicated that the sample achieved 80% power to detect effects (Cohen's *w*) up to 0.11. The effects observed for low morality (*w* = 0.15, χ²=17.5, *p*<.001) and low sociability (*w* = 0.13, χ²=13.5, *p* =.001) both exceed this threshold.

Sensitivity analysis for one-way ANOVA indicated that the sample had 80% power to detect effects up to *f* = [.20], as in the case of the effect observed for differences between groups regarding morality (*f* = 0.23, partial η² = .051).

# Supplementary Figures and Tables

*Content warning: Please note that this section contains language that some readers may find upsetting.*

**Table 1.** Unmatched words qualitative coding

| **Word** | **Frequency** | **Category** |
| --- | --- | --- |
| billy | 1 | ability low & negative |
| boor | 2 | sociability low & negative |
| calafrican | 1 | geography/social group |
| camorra | 1 | morality low & negative |
| copious | 1 | other |
| emigrant | 1 | social group |
| energic | 1 | assertiveness high & positive |
| evader | 1 | morality low & negative |
| food | 1 | other |
| glutton | 2 | other |
| hothead | 1 | morality low & negative |
| intrusive | 5 | morality low & negative |
| latecomer | 3 | morality low & negative |
| mafia | 32 | morality low & negative |
| muddler | 1 | morality low & negative |
| naples | 4 | geography/social group |
| nitwit | 1 | ability low & negative |
| parasite | 3 | morality low & negative/assertiveness low & negative |
| profiteer | 10 | morality low & negative |
| scugnizzi | 1 | other |
| shepherd | 4 | occupation |
| sicily | 1 | geography/social group |
| tax evader | 1 | morality low & negative |
| terrone | 92 | ability low & negative |
| underdeveloped | 1 | status low & negative/ability low & negative |
| united | 1 | sociability high & positive |
| unregulated | 1 | morality low |
| unserious | 1 | morality low & negative |
| welfarism | 1 | morality low |

Note. “Calafricani”: local or colloquial dialect expression combining “Calabria” with “Africa” referred to the residents of the Calabria region, indicating the proximity between southern Italy and the African continent; “Camorra”: mafia association originated in the Campania region; “Terrone”: term employed in a derisory or humorous manner to denote the residents of southern Italy, historically associated with agriculture and cultural and educational lack. “Scugnizzi”: colloquial term referred to young people hailing from the Neapolitan street.

environment.

**Table 2.** Most frequent words for each category

| **Word** | **Frequency** | **Percentage** | **Category** | **North** | **Center** | **South** |
| --- | --- | --- | --- | --- | --- | --- |
| terron* | 92 | 11.2 % | ability | 24 | 33 | 35 |
| ignorant | 40 | 4.9% | ability | 6 | 10 | 24 |
| backward | 16 | 1.9% | ability/beliefs | 0 | 6 | 10 |
| lazybones | 26 | 3.2% | assertiveness | 9 | 9 | 8 |
| lazy | 21 | 2.6% | assertiveness | 8 | 2 | 11 |
| southerner | 20 | 2.4% | geography | 11 | 5 | 4 |
| mafia | 32 | 3.9% | morality | 10 | 10 | 12 |
| generous | 17 | 2.1% | morality | 4 | 3 | 10 |
| sly | 16 | 1.9% | morality | 5 | 5 | 6 |
| thief | 14 | 1.7% | morality | 3 | 8 | 3 |
| idle | 30 | 3.7% | morality/assertiveness | 10 | 14 | 6 |
| warm | 47 | 5.7% | sociability | 11 | 13 | 23 |
| welcoming | 40 | 4.9% | sociability | 8 | 14 | 18 |
| cheerful | 27 | 3.3% | sociability | 3 | 10 | 14 |
| hospitable | 21 | 2.6% | sociability | 6 | 7 | 8 |
| sociable | 16 | 1.9% | sociability | 4 | 5 | 7 |
| nice | 16 | 1.9% | sociability | 5 | 6 | 5 |
| poor | 15 | 1.8% | status | 9 | 3 | 3 |

**Table 3.** Words coded in multiple categories due to dimensional overlapping

| **Word** | **Frequency** | **Category** |
| --- | --- | --- |
| disadvantaged | 1 | morality & status |
| dull | 1 | sociability & ability |
| good life | 1 | morality, ability & assertiveness |
| good natured | 3 | sociability & morality |
| helpful | 2 | sociability & morality |
| hostile | 1 | sociability & morality |
| idle | 30 | morality & assertiveness |
| ignored | 1 | ability & status |
| impaired | 1 | morality & ability |
| kind | 2 | sociability & morality |
| modest | 1 | morality & status |
| parasite | 3 | morality & assertiveness |
| positive | 1 | sociability & assertiveness |
| rough | 1 | sociability & ability |
| rowdy | 8 | sociability & morality |
| self-pitying | 1 | sociability & status |
| social butterfly | 1 | sociability & morality |
| strong | 1 | assertiveness & status |
| underdeveloped | 1 | ability & status |
| violent | 1 | morality & assertiveness |
